# Supplementary material for: Implementation of RT-RAA and CRISPR/Cas13a for an NiV Point-of-Care Test: A Promising Tool for Disease Control
Source: Viruses. 2025 Mar 27;17(4):483. doi: 10.3390/v17040483 (PMC12031521; doi:10.3390/v17040483)
Supplement: Supplementary file 1 [file viruses-17-00483-s001.zip › viruses-3466282-supplementary.pdf]

Supplementary Information

Implementation of RT-RAA and CRISPR/Cas13a for an NiV Point-of-Care Test: A Promising Tool for Disease Control

Authors:

Jingqi Yin [1,2], Jin Cui [2], Hui Zheng [2], Tingting Guo [3], Rong Wei [2], Zhou Sha [2*], Shaopeng Gu [1*] and Bo Ni [1,2*]

Affiliations:

1 Shanxi Agricultural University, Jinzhong 030031, China;

2 China Animal Health and Epidemiology Center, Qingdao266011, China;

3 Yangzhou University, Yangzhou 225001, China

* Correspondence: Zhou Sha* shazhou@cahec.cn, Shaopeng Gu* shpgu@163.com, Bo Ni* nibo@cahec.cn

Table S1. NCBI reference sequence of the NiV complete genome.

Definition	NCBI Reference Sequence
Nipah virus, complete genome	NC_002728.1
Nipah virus, complete genome	AF212302.2
Nipah virus isolate NIVBGD2008RAJBARI, complete genome	JN808863.1
Nipah virus isolate NIVBGD2008MANIKGONJ, complete genome	JN808857.1
Nipah virus isolate Ind-Nipah-07-FG from India, complete genome	FJ513078.1
Nipah virus isolate UMMC2, complete genome	AY029768.1
Nipah virus isolate UMMC1, complete genome	AY029767.1
Nipah virus from Bangladesh, complete genome	AY988601.1
Nipah virus complete genome, isolate NV/MY/99/VRI-0626	AJ627196.1
Nipah virus complete genome, isolate NV/MY/99/UM-0128	AJ564623.1
Nipah virus complete genome, isolate NV/MY/99/VRI-1413	AJ564622.1
Nipah virus complete genome, isolate NV/MY/99/VRI-2794	AJ564621.1
Henipavirus nipahense strain MCL-18-H-1088, complete genome	MH523642.1
Henipavirus nipahense isolate NiV/TH/P.lylei/2017/B17640.GUL, complete genome	MW535746.1
Henipavirus nipahense isolate 201601241, complete genome	MK673592.1
Henipavirus nipahense isolate 201601231, complete genome	MK673591.1
Henipavirus nipahense isolate 201601191, complete genome	MK673590.1
Henipavirus nipahense isolate 201601187, complete genome	MK673589.1
Henipavirus nipahense isolate 201601144, complete genome	MK673587.1
Henipavirus nipahense isolate 201601138, complete genome	MK673586.1
Henipavirus nipahense isolate 201601136, complete genome	MK673585.1
Henipavirus nipahense isolate 201601126, complete genome	MK673584.1
Henipavirus nipahense isolate 201302591, complete genome	MK673583.1
Henipavirus nipahense isolate 201206325, complete genome	MK673582.1
Henipavirus nipahense isolate 201206316, complete genome	MK673581.1
Henipavirus nipahense isolate 201206312, complete genome	MK673579.1
Henipavirus nipahense isolate 201206119, complete genome	MK673578.1
Henipavirus nipahense isolate 201206008, complete genome	MK673577.1
Henipavirus nipahense isolate 201200973, complete genome	MK673576.1
Henipavirus nipahense isolate 201200938, complete genome	MK673575.1
Henipavirus nipahense isolate 201200920, complete genome	MK673574.1
Henipavirus nipahense isolate 201200919, complete genome	MK673573.1
Henipavirus nipahense isolate 201200903, complete genome	MK673571.1

Henipavirus nipahense isolate 812001, complete genome	MK673570.1
Henipavirus nipahense isolate 811373, complete genome	MK673568.1
Henipavirus nipahense isolate 810482, complete genome	MK673567.1
Henipavirus nipahense isolate 810428, complete genome	MK673566.1
Henipavirus nipahense isolate 810405, complete genome	MK673565.1
Henipavirus nipahense isolate 810398, complete genome	MK673564.1
Henipavirus nipahense isolate 808652, complete genome	MK673563.1
Henipavirus nipahense isolate 808651, complete genome	MK673562.1
Henipavirus nipahense isolate 808589, complete genome	MK673561.1
Henipavirus nipahense isolate 808585, complete genome	MK673560.1
Henipavirus nipahense isolate 808581, complete genome	MK673559.1
Henipavirus nipahense isolate 808579, complete genome	MK673558.1
Henipavirus nipahense isolate CSUR381, complete genome	MK801755.1
Henipavirus nipahense strain MCL-18-H-1209, complete genome	MH523641.1
Henipavirus nipahense strain MCL-18-H-1197, complete genome	MH523640.1
Henipavirus nipahense strain MCL-18-H-1088, complete genome	MH396625.1
Henipavirus nipahense isolate 201206313, complete genome	MK673580.1
Henipavirus nipahense isolate MCL-19-BAT-572-7, complete genome	MN549409.1
Henipavirus nipahense strain NiV/BD/P.medius/EHA/2013/Sylhet191, complete genome	MK575070.1
Henipavirus nipahense strain NiV/BD/P.medius/EHA/2013/Raypur1411, complete genome	MK575069.1
Henipavirus nipahense strain NiV/BD/P.medius/EHA/2013/Raypur1410, complete genome	MK575068.1
Henipavirus nipahense strain NiV/BD/P.medius/EHA/2013/Raypur1409, complete genome	MK575067.1
Henipavirus nipahense strain NiV/BD/P.medius/EHA/2013/Raypur1408, complete genome	MK575066.1
Henipavirus nipahense strain NiV/BD/P.medius/EHA/2013/Raypur1406, complete genome	MK575065.1
Henipavirus nipahense strain NiV/BD/P.medius/EHA/2013/Raypur1405, complete genome	MK575064.1
Henipavirus nipahense strain NiV/BD/P.medius/EHA/2013/Raypur1404, complete genome	MK575063.1
Henipavirus nipahense strain NiV/BD/P.medius/EHA/2013/Raypur1403, complete genome	MK575062.1

Henipavirus nipahense strain NiV/BD/P.medius/EHA/2013/Raypur1402, complete genome	MK575061.1
Henipavirus nipahense strain NiV/BD/P.medius/EHA/2013/Raypur1401, complete genome	MK575060.1

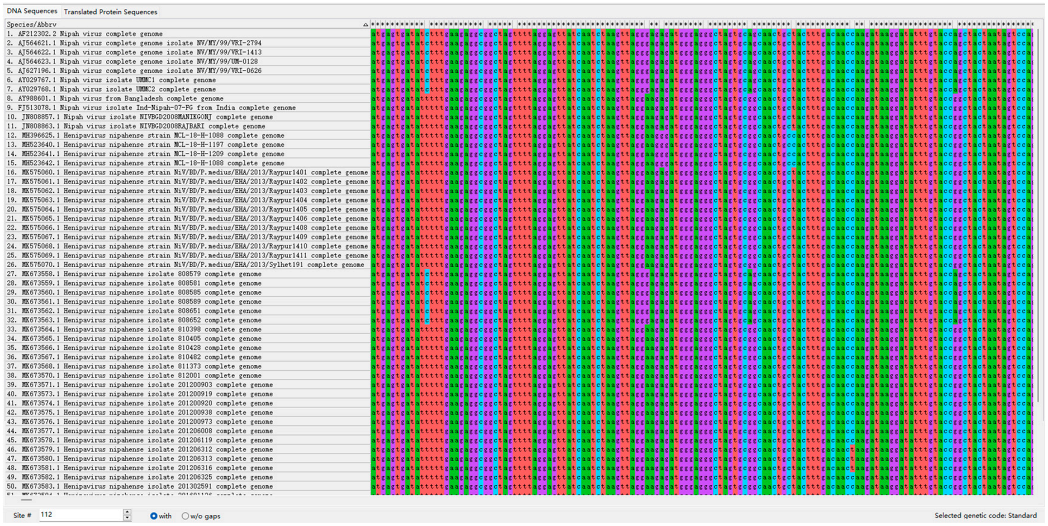

Figure S1. Sequence alignment results.

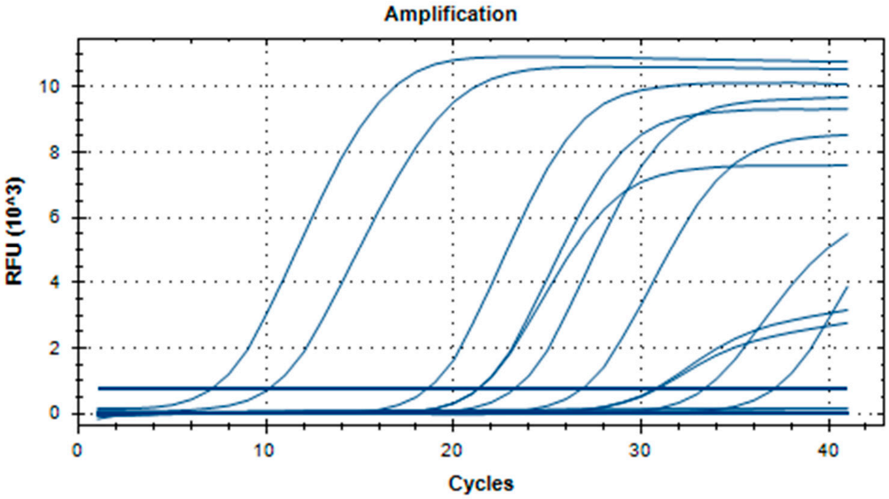

Figure S2. RT-qPCR results. The RT-qPCR results show that 11 curves, corresponding to 10 positive simulated NiV clinical samples and one positive control, exhibit clear amplification peaks, indicating the presence of NiV ssRNA template. These samples have low CT values, suggesting a higher template concentration. In contrast, both the negative control and negative simulated NiV clinical samples show no amplification, confirming the specificity of the test and the absence of NiV ssRNA template in these samples.
